# OMRT-5. Therapy-induced reprogramming drives glioma vascular transdifferentiation and recurrence

**DOI:** 10.1093/noajnl/vdab070.030

**Published:** 2021-07-05

**Authors:** Sree Deepthi Muthukrishnan, Riki Kawaguchi, Pooja Nair, Rachna Prasad, Yue Qin, Michael Condro, Qing Wang, Alvaro Alvarado, Harley Kornblum

**Affiliations:** University of California, Los Angeles, CA, USA

## Abstract

Therapy-resistant glioma cells elicit remarkable phenotypic plasticity leading to aggressive tumor recurrence. Here, we used single-cell and whole transcriptomic sequencing to uncover that radiation treatment induces a dynamic shift in functional states of glioma cells allowing for acquisition of either stem-like, mesenchymal-like or vascular-like phenotypes. The predominant phenotype switch induced by radiation in surviving tumor cells is the vascular-like cell state, resulting in transdifferentiation to endothelial-like and pericyte-like cells in distinct cell clusters. The transdifferentiated endothelial-like and pericyte-like cells secrete trophic factors to support proliferation of tumor cells, and their selective ablation results in reduced tumor growth and recurrence post-treatment. Mechanistically, the acquisition of vascular-like phenotype is driven by increased acetylation and chromatin accessibility in vascular genes and in regions for binding of vascular specification transcription factors. Blocking histone acetylation using a small molecule inhibitor targeting P300 histone acetyltransferase activity prior to radiation treatment inhibits the vascular-like transdifferentiation of glioma cells and tumor growth. Our findings indicate that radiation therapy-induces rewiring of glioma cells that promotes vascular cell-like transdifferentiation, tumor growth and recurrence.

